# Environmental implications and recovery potential of rare earth elements in solid residues from the incineration of sewage sludge

**DOI:** 10.1038/s41598-025-32315-0

**Published:** 2025-12-16

**Authors:** Bartłomiej Michał Cieślik, Oskar Ronda, Satoki Okabayashi, Koichi Chiba, Motohiro Tsuboi, Justyna Płotka-Wasylka

**Affiliations:** 1 Faculty of Chemistry, Department of Analytical Chemistry, Gdańsk University of Technology, Gabriela Narutowicza 11/12 str, 80-233 Gdańsk, Poland; 2 Department of Applied Chemistry for Environment, School of Biological and Environmental Sciences, Kwansei Gakuin University, 1 Gakuen Ugahara, Sanda, 669-1330 Japan

**Keywords:** Fluidized bed, Rare earth elements, Recovery, Sewage sludge ash, Trace analysis, Waste management, Environmental sciences, Chemistry, Analytical chemistry

## Abstract

The presented research aimed to contribute new insights into the chemical composition of solid waste generated during the incineration of sewage sludge, with a particular focus on the occurrence and behaviour of rare earth elements (REEs). In this study, the total content as well as the mobility of rare earth elements (REE) were determined in disposed fluidized beds (FB), sewage sludge ash (SSA), and air pollution control residues (APC) samples obtained from three Polish facilities engaged in thermal sewage sludge treatment. The mobility of REE was assessed using the BCR sequential extraction procedure. Each type of tested material was characterized by different content of REE (SSA > FB > APC). Moreover, statistically significant differences were observed in the REE content in individual waste fractions depending on the sampling places. The mobility of REE in SSA and FB was determined as very low (approx. 98.5% of Sc and > 99% of other REE in the immobile fraction). Based on the results, it can be concluded that the recovery potential of REE from each fraction of the tested waste is negligible. Because of the very low mobility of REE, these elements should not be considered hazardous during FB and SSA management.

**Supplementary Information:**

The online version contains supplementary material available at 10.1038/s41598-025-32315-0.

## Introduction

 Rare earth elements (REE) are a term describing a group of elements that includes lanthanides, scandium (Sc), and yttrium (Y). In addition to traditional applications, such as production of the polishing agents or additives to glass, these elements are widely used in many high-tech industry products. Popular applications of REE include the production of lasers, consumer electronics, special-purpose metallic alloys, catalysts, magnets, and chips^1^. Meeting the growing technological needs of civilization results in a constantly growing demand for REE, which is becoming increasingly difficult to meet through conventional mining of the mentioned elements. In addition, REE mining carries the risk of contamination of the nearby environment with rare earth metals^2^ and the occurrence of health complications among miners and residents of areas located near mines, not only due to contamination of their bodies with REE but also to co-occurring heavy metals, including radionuclides^3^. Furthermore, the processing of REE ores generates a number of hazardous wastes and greenhouse gases^4^.

For economic reasons, as well as taking into account concern for the state of the natural environment and the health of the population, alternative sources of REE are currently being sought. The most promising sources of REE include several waste materials, including electronic waste containing permanent magnets, which typically contain 27–31% of REE^5^ or coal ash, in which the average content of REE around the world is estimated at 404.50–435.45 mg/kg^6,7^. Sewage sludge ash (SSA) is of less interest in the context of REE recovery, due to the (usually) lower content of these elements in its composition, compared to the previously mentioned ashes from coal combustion^8–10^. However, it should be remembered that although SSA is the majority of the waste fraction in the process of sewage sludge incineration in fluidized bed furnaces, it is not the only such fraction. The others waste fractions (air pollution control residues and fluidized beds) are poorly researched and, to the authors’ knowledge, have not yet been characterized in terms of the content of rare earth metals. The presence of REE in waste resulting from the incineration of sewage sludge may not only be an opportunity for its recovery but also an obstacle to the management of this waste. A particularly high risk is associated with the potential leaching of REE from the mentioned materials into the soil, which may change its chemistry. This risk may potentially arise in the case of several possible SSA management methods described in the literature, in particular in the case of using phosphorus fertilizers produced based on SSA^11–14^ or building materials doped with SSA^15–17^, as well as in the case of using SSA as a land reclamation agent^18^.

The environmental risk associated with potentially toxic metals also occurs when the waste in question is stored in non-hazardous waste landfills (SSA and spent fluidized beds are stored in the mentioned type of landfills). The leaching of metals from SSA has been the subject of research by many research teams, which confirmed the diverse mobility of metals^14,19–21^. For example, the results of the experiment using the BCR sequential extraction method show that elements like Bi, Cr and Pb are present in SSA almost exclusively in the considered as immobile residual fraction, while more mobile forms of Rb, Sr, Ag, V, Cd, Co constitute 20–30% of the total content of mentioned elements^21^. To the authors’ knowledge, however, such analyses taking into account REE have not been carried out yet.

The aim of this research is to provide new insights into the occurrence, distribution, and mobility of rare earth elements (REEs) in three distinct solid residues generated during the incineration of sewage sludge in fluidized bed furnaces: sewage sludge ash (SSA), air pollution control residues (APC), and spent fluidized beds (FB). The study focuses on determining the content and performing fractionation of REEs using the BCR sequential extraction procedure to evaluate their environmental mobility. Based on the obtained results, the potential of these residues to serve as alternative secondary sources of REEs is assessed, together with an evaluation of the potential environmental risks associated with the leaching of these elements from the tested materials. The innovative aspect of this study lies not only in the simultaneous characterization of waste fractions such as SSA, APC, and FB that, to the authors’ knowledge, have not previously been examined together in the context of REE behaviour, but also in the comparative approach that provides new insight into REE partitioning mechanisms during sewage sludge incineration. By evaluating how REEs distribute among different solid residues and how their mobility varies across operationally defined fractions, this study offers a deeper understanding of the processes governing their stabilization or potential release. Such knowledge is essential both for assessing the realistic recovery potential of REEs from these materials and for improving environmental risk management strategies related to the handling, storage, and possible reuse of sewage sludge incineration residues.

## Methods

### Research material

**Fig. 1:**
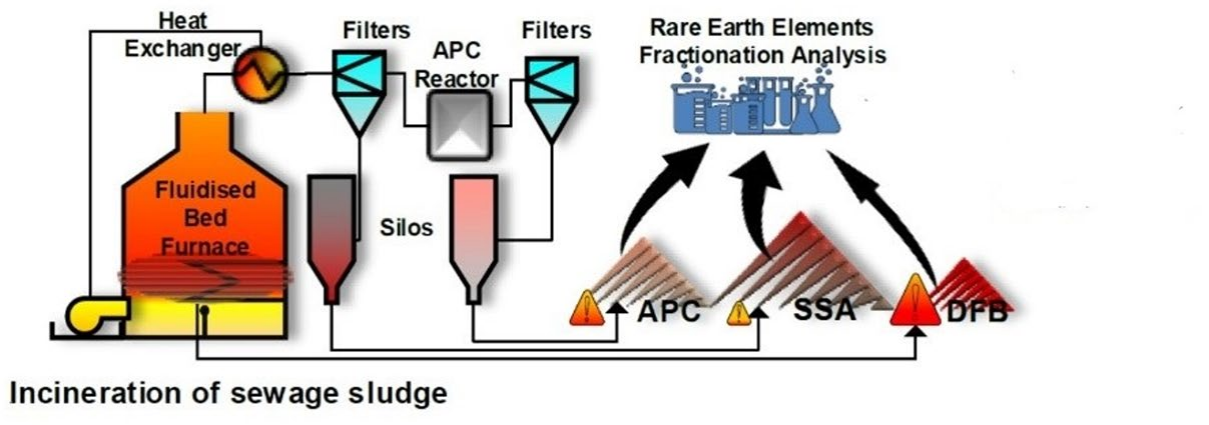
Simplified scheme of the sewage sludge incineration plant with marked waste fractions subject to analysis.

The research material comprises samples from three waste fractions: sewage sludge ash (fly ash) (SSA), air pollution control residues (sorbents) (APC), and fluidized bed (FB) residues generated during the combustion of sewage sludge in fluidized bed furnaces. The elements of the industrial installation from which individual waste fractions were collected are marked in the drawing presented in the Fig. 1. These samples, spanning the years 2014 to 2021, were gathered from three distinct facilities engaged in the thermal processing of sewage sludge situated in Poland—specifically, Gdańsk Wschód, GOŚ Łódź, and GOŚ Dębogórze (Table 1). These facilities receive sewage sludge from municipal sewage treatment plants located in major urban agglomerations. The estimated daily sewage flows for the respective facilities are as follows: GOŚ Łódź − 210,000 m^3^/d, Gdańsk Wschód − 95,000 m^3^/d, GOŚ Dębogórze − 55,000 m^3^/d. In total, 17 SSA samples, 12 APC samples, and 4 FB samples were collected for analysis.

**Table 1:** The detailed information on sample origin.

Sampling place	Sewage sludge ash (SSA) samples	Air pollution control system residues (APC) samples	Fluidized bed samples (FB)
“Gdańsk Wschód” Acronym: Gda	Sample 1: July 2014 Sample 2: November 2015 Sample 3: November 2016 Sample 4: September 2018 Sample 5: February 2019 Sample 6: March 2021 Sample 7: June 2021	Sample 1: November 2015 Sample 2: April 2016 Sample 3: November 2016 Sample 4: September 2018 Sample 5: February 2019 Sample 6: November 2019 Sample 7: June 2021	Sample 1: September 2018 Sample 2: June 2021
“GOŚ Łódź” Acronym: Lod	Sample 1: June 2015 Sample 2: November 2015 Sample 3: April 2016 Sample 4: April 2016 Sample 5: July 2016 Sample 6: May 2018 Sample 7: February 2021	Sample 1: November 2015 Sample 2: February 2016 Sample 3: April 2016 Sample 4: July 2016 Sample 5: February 2021	Sample 1: June 2018
“GOŚ Dębogórze” Acronym: Gdy	Sample 1: June 2016 Sample 2: March 2021 Sample 3: June 2021	no samples collected	Sample 1: June 2021

### Quantification of rare Earth elements (REE)

The pseudo-total content of selected trace elements in SSA, APC, and FB was determined, as well as the share of individual fractions of these elements, separated based on their mobility in the environment, in SSA and FB. In this research, the pseudo-total content is defined as the amount of REE in tested materials that can be dissolved by microwave-assisted digestion in aqua regia and it does not contain REE permanently associated with the faction the silica fraction (soluble in mixtures containing hydrofluoric acid). The silica fraction was not determined because it is insoluble in media other than HF, thus it is negligible in the context of estimating the REE recovery potential or environmental risk. Determination of the pseudo-total content of REE in tested materials was performed using the following procedure. The samples were digested in aqua regia (10 ml of aqua regia per 0.5 g of sample) using microwave-assisted mineralization (200 °C, 50 min). After that 1 ml of a sample was evaporated (80 °C, 3 h) and diluted in 10 ml of 2% HNO_3_ solution. To dissolve all residues after evaporation, all samples were treated in ultrasound bath for 20 min and heated at 80 °C for 20 min. Metal fractionation was performed using a modified BCR sequential extraction model^22^. The tests used twice smaller amounts of starting material (0.5 g) and twice smaller amounts of reagents compared to the cited procedure. This model allows for the determination of four fractions of each element: exchangeable and carbonates fraction (F1), reducible fraction (F2), oxidizable fraction (F3), and residual fraction (F4). Fraction F1 is the most mobile fraction, so it is potentially the most hazardous and impactful fraction on the environment, while the F4 fraction is considered stable. The content of REE in extracts and solutions obtained as a result of digestion was determined using the Inductively Coupled Plasma - Mass Spectrometer (ICP-MS) model, 7700 Agilent Technologies. All reagents used for samples and calibration preparation were ICP purity. The measurement uncertainties estimated on the basis of the relative standard deviation are: 0.19–13% Sc; 0.15–2.0% Y; 0.70–2.0% La; 0.019–1.1% Ce; 0.17–2.5% Pr; 0.010–7.2% Nd; 0.25–7.8% Sm; 0.13–12% Eu; 0.049–7.8% Gd; 0.21–8.4% Tb; 0.074–6.1% Dy; 0.12–6.2% Ho; 0.24–8.7% Er; 0.16–21% Tm; 0.032–6.6% Yb and 0.17–10% Lu. The validation parameters of the analytical methods used in the study are presented in Table S1 in the Supplementary Information. All element content measurements were preceded by blank correction.

### Presentation of results of analysis

The results of the pseudo-total content of REE determination as well as the fractionation of these elements, were presented on the box plots in which the median value is marked as a horizontal line inside the box and the average value is marked as an “x” symbol. The Eu anomaly was calculated using Eq. (1)^23^.

1 $${\mathrm{Eu}}_{{\mathrm{anom}}}={\mathrm{Eu}}_{{\mathrm{N}}}/({\mathrm{Sm}}_{{\mathrm{N}}}\cdot {\mathrm{Gd}}_{{\mathrm{N}}})^{0.5}$$

where Eu_N_, Sm_N_, Gd_N_ are normalized concent of Eu, Sm and Gd, respectively. The Post-Archean Australian Shale (PAAS) normalization standard was used for the calculation^24^.

## Results and discussion

**Fig. 2:**
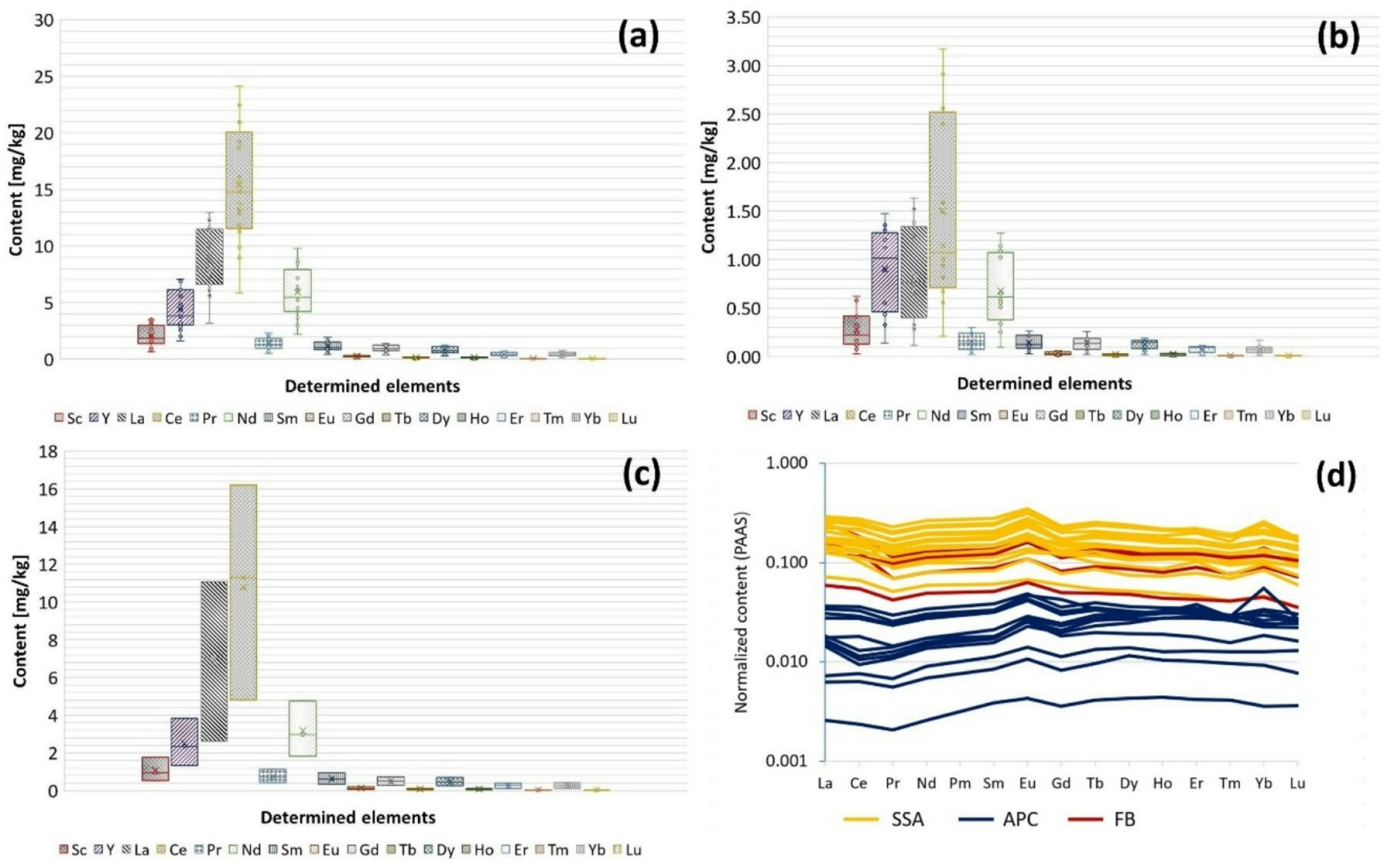
The pseudo-total content of REE in tested fractions of waste generated in sewage sludge combustion process (**a** SSA, **b** APC, **c** FB) and values normalized to the REE content in the Post-Archean Australian Shale (**d**)^27^.

The detailed results of REE determination in the samples are included in Table S2 and Table S3 in the Supplementary Information. The pseudo-total content of REE in the studied materials is placed in the range: 15.6–65.8 mg/kg SSA; 12.9–42.6 mg/kg FB and 0.723–9.1 mg/kg APC. These values are significantly lower compared to the content of REE in more promising sources of the mentioned elements, such as coal ash^25,26^. The dominant rare earth elements in all fractions of the examined waste materials are Ce, La, Nd and Y (Fig. 2). The content of critical REE (Y, Nd, Eu, Tb, Dy, Er) was calculated in relation to the content of excessive REE (Ce, Ho, Er, Yb, Lu) - the outlook coefficient (C_outl_). The calculated values of the outlook coefficient are: C_outl_ SSA = 0.74; C_outl_ APC = 1.32; C_outl_ FB = 0.78. In the case of SSA and FB, these values ​​are ​​close to the lower limit of C_outl_ values for REE-rich coal ash, which is considered promising in terms of REE recovery (0.7 ≤ C_outl_ ≤ 1.9)^27^. Compared to SSA, one order of magnitude lower contents of the elements in question were recorded in APC (Fig. 2b), therefore this waste fraction cannot be considered as promising source of REE although the highest C_outl_ value among all studied materials. Approximately 1.5–2 times lower contents of individual REEs in comparison with SSA were recorded in fluidized beds (Fig. 2c). The exception is yttrium (Y), which relative content (compared to the content of other tested elements) in the APC fraction is slightly higher than in the case of other materials. In the case of the tested samples, the content of individual lanthanides in the APC fraction is usually two orders of magnitude lower, and in the SSA and FB fractions one order of magnitude lower than the content of these elements in Post-Archean Australian Shale (Fig. 2d)^27^. The normalization presented in Fig. 2d was applied to identify possible anomalies and assess whether the REE distribution reflects natural background levels or potential anthropogenic influence. The Post-Archean Australian Shale (PAAS) is commonly used in geochemistry as a reference standard because it represents the average composition of the upper continental crust. Normalisation to PAAS therefore, allows a direct comparison between waste-derived residues and natural background levels. A flat pattern indicates a composition consistent with natural crustal material, while upward or downward deviations suggest enrichment or depletion of specific REEs, potentially linked to anthropogenic contributions or geochemical fractionation processes. It can be observed that the ratio of the content of individual lanthanides in the tested materials to the content of these elements in the Post-Archean Australian Shale is approximately constant, which suggests the dominant share of natural sources of these elements in municipal sewage sludge. Small, positive anomalies were observed in the case of europium (Eu) and ytterbium (Yb), which have no practical significance when discussing the possibilities and risks related to the management of the tested materials. The Eu anomaly calculated based on Eq. 1 is very similar for every fraction of waste − 1.285 ± 0.078 for SSA, 1.306 ± 0.036 for APC and 1.314 ± 0.058 for FB. The level of positive Eu anomaly slightly depends on the sampling place − 1.280 ± 0.064, 1.360 ± 0.040, 1.245 ± 0.067 for samples from Gdańsk Wschód, GOŚ Łódź, and GOŚ Dębogórze thermal treatment plants, respectively. The nature of the Eu positive anomaly is difficult to explain, however, it is known that wastewater is characterized by an Eu positive anomaly, often^23^. This element is removed from wastewater in a sewage treatment plant and ends up in the sludge. Wastewater is often characterized by the presence of a strong Gd-anomaly^28–30^. This phenomenon has anthropogenic origins and is mainly related to MRI contrast agents found in sewage^31,32^. However, previous studies indicate the absence or presence of very weak Gd-anomalies in sewage sludge^28,33^. The presented research did not demonstrate the presence of Gd-anomaly in any fraction of solid waste generated in the process of sewage sludge incineration.

**Fig. 3:**
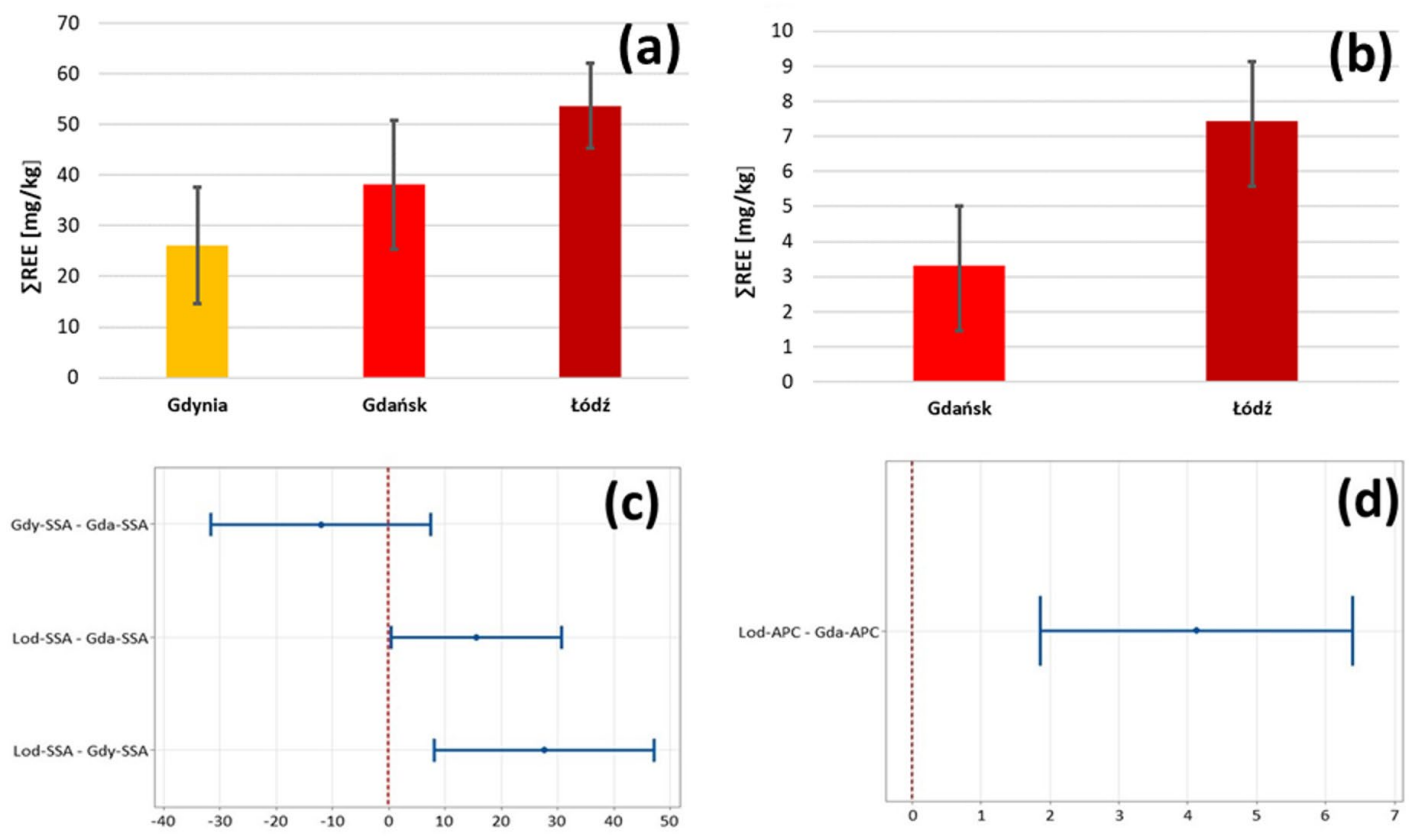
The distribution of the summary content of rare earth metals (REE) in samples by sampling place (average ± SD; **a** SSA samples; **b** APC samples) and tukey’s test results (**c** SSA samples; **d** APC samples).

The distribution of the summary content of rare earth metals (REE) in samples by type and place of origin is presented in Fig. 3a,b. To determine whether the samples differ statistically depending on the place of origin, a one-way ANOVA was performed. It was shown that in the case of both SSA and APC samples, there was a statistically significant correlation between the sampling place and the total REE content (α = 0.05; p-values 0.005 and 0.002, respectively, for the null hypothesis regarding equality of groups). FB samples were omitted from the analysis due to their insufficient quantities. The tukey’s post hoc test showed that in the case of SSA, one group consists of samples from Gdynia and Gdańsk (no statistically significant differences between them, probably because of close vicinity of both facilities) and the second group consists of samples from Łódź, characterized by statistically significantly higher REE contents (Fig. 3c). In the case of APC samples, the existence of two separate groups was also demonstrated - samples from Gdańsk and Łódź are statistically different from each other (Fig. 3d). Since, regardless of the place of origin of the samples, no anomalies in relation to the natural REE ratio are observed (Post-Archean Australian Shale), it can be concluded that the differences in the total REE content between sampling places are of natural origin.

**Fig. 4:**
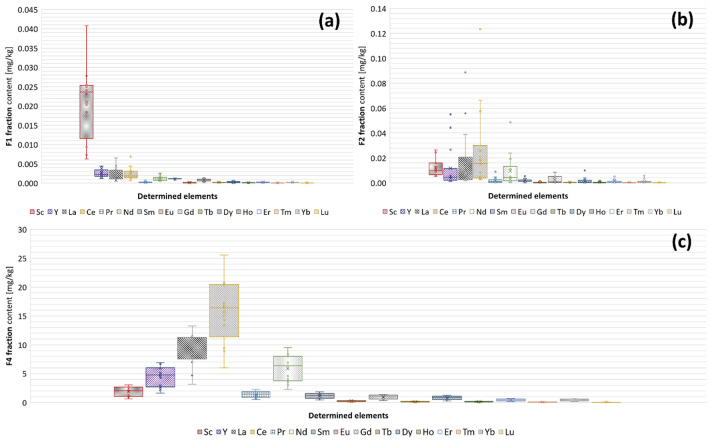
The fractionation results of REE in tested samples of sewage sludge ash (SSA) and fluidized beds (FB). (**a**) Exchangeable and carbonates fraction (F1); (**b**) reducible fraction (F2); (**c**) residual fraction (F4). The content of the oxidizable fraction (F3) in the studied samples was below the LOQ.

Figure 4 shows the results of REE fractionation in SSA and FB. It was decided to present the fractionation results jointly for SSA and FB, due to the very similar distribution of REE determination results in individual extracts obtained from both types of tested materials. Fractionation of the tested elements in APC samples was abandoned due to the very high content of strongly alkaline substances used as a sorbent (mainly calcium carbonate and sodium carbonate), which prevents the use of the BCR sequential extraction technique, as well as the low REE content in these materials. It was observed that all rare earth elements occur in SSA and FB are bound to the residual fraction (F4) almost exclusively, which is considered an immobile fraction. In the case of scandium (Sc), a slightly higher share of the F1 fraction was observed compared to the remaining REEs, although the F4 fraction is still the dominant fraction (approx. 98.5% share). In the case of other light rare earth metals (LREE – Ce, Pr, Nd, Sm, Eu), the share of the F4 fraction always exceeded 99% by mass. Determining the percentage of mobile fractions in heavy rare earth metals (HREE –Gd, Tb, Dy, Ho, Er, Tm, Yb, Lu) is subject to high uncertainty due to the low content of these elements in the tested materials. However, as in the case of LREE, it can be stated that the F4 fraction is dominating. The low mobility of rare earth metals in SSA and FB is related to the formation of highly stable mineral structures under combustion conditions. However, to describe the composition of mineral structures containing REEs included in SSA and FB, it is necessary to perform additional research. To the author’s knowledge, there is only one report about the mineral structure of REE in SSA, however, the results of our research do not seem to confirm the thesis that Y occurs in the form of carbonates^34^. Differences in theses may result from the use of ash generated after combustion of sewage sludge in a laboratory furnace in the cited work, and not obtained using a fluidized bed furnace. Significantly different combustion conditions may lead to products with different REE speciation characteristics and this phenomenon was confirmed by comparison of the mobility of metals in laboratory-made SSA and industrial origin one^35^. In particular, the lack of intensive mixing of the material during combustion in laboratory furnaces (contrary to fluidized bed furnace combustion) may result in incomplete removal of carbon from the sample, and some carbonates can remain in the sample. It should be noted, that at the time of writing this work, only coal combustion residues are well characterized concerning the mobility of rare earth elements. Compared to SSA and FB, coal residues are characterized by a lower percentage of immobile fraction, generally described as a residual fraction, determined by using BCR sequential extraction or similar procedures, which are 70–99%^36^; 50–99%^37^; 60–85%^38^. There is also a single report in the literature about the speciation of rare earth elements in medical waste incinerator ash^39^. The cited research shows that in this type of waste, the typical content of stable, residual fraction of REE is about 60–80% which is significantly less compared to the content of this fraction in tested waste materials from the sewage sludge combustion process. Based on the analysis of research data and literature, it can be concluded that REE contained in SSA and FB are expected to pose a lower environmental risk compared to REE present in coal ashes or medical waste incinerator ashes. The low mobility of REE means a limited risk to the environment related to the leaching of the discussed elements from the tested materials during their management (e.g. as a means of land reclamation). On the other hand, the dominant share of the residual fraction (F4) leads to the conclusion that REEs are relatively permanently immobilized in SSA and FB and are resistant to leaching using mild acidic conditions or in reducing and oxidizing environments. This state means that an effective and economically justified method of recovering REE from sewage sludge ash (even in the case of the high content of these elements in SSA or FB) by leaching seems to be difficult to develop.

## Conclusion

Analysing the content of REE in tested waste materials leads to the conclusion that solid waste from the incineration of sewage sludge from municipal sewage treatment plants is not a source of REE that could be used for the economically justified recovery of these valuable elements. Possible considerations regarding the recovery of REE from the described materials should therefore be limited to specific cases of SSA generated from the combustion of certain industrial sewage sludges, where a significantly higher REE content could be expected. However, such cases require further targeted investigation, as they were not within the scope of this study. The mentioned SSA may be characterized by a significantly higher content of lanthanides (non-regular sewage sludge combustion) only in cases of some specific industries. The environmentally crucial conclusion is that the rare earth metals contained in sewage sludge ash and fluidized beds are highly immobilized, thus not posing a threat to the environment by leaching the mentioned elements from the tested materials during storage or management. It should also be noted that the possibility of economically justified recovery of REE depends not only on their concentration and chemical form, but also on the volume and mass of waste generated in sewage sludge incineration plants, as well as on process-related costs. These aspects were beyond the scope of the present work, but they constitute an essential direction for future studies aiming at a comprehensive evaluation of recovery potential. The authors note the scope for future research, which should be to learn the detailed mineralogical structure and chemical combinations of REE occurring in solid waste after sewage sludge incineration.

## Supplementary Information

Below is the link to the electronic supplementary material.

Supplementary Material 1

## Data Availability

The datasets generated during and/or analysed during the current study are available from the corresponding author on reasonable request while the crucial data are added in supplementary materials.
