# Supplementary material for: Environmental implications and recovery potential of rare earth elements in solid residues from the incineration of sewage sludge
Source: Sci Rep. 2025 Dec 16;16:2550. doi: 10.1038/s41598-025-32315-0 (PMC12820271; doi:10.1038/s41598-025-32315-0)
Supplement: Supplementary file 1 — Supplementary Material 1 [file 41598_2025_32315_MOESM1_ESM.docx]

Table S1. Validation parameters of analytical methods used in the study

| Isotope | Calibration function | R^2^ | LOD [µg/kg] | LOQ [µg/kg] |
| --- | --- | --- | --- | --- |
| ^45^Sc | y = 1108c + 170.5 | 0.9988 | 0.34 | 1.0 |
| ^89^Y | y = 6668c + 737.6 | 0.9988 | 0.23 | 0.70 |
| ^139^La | y = 16860c + 1100 | 0.9989 | 0.25 | 0.76 |
| ^140^Ce | y = 21410c + 1876 | 0.9988 | 0.11 | 0.34 |
| ^141^Pr | y = 26630c + 2104 | 0.9988 | 0.18 | 0.54 |
| ^143^Nd | y = 3090c + 272.8 | 0.9987 | 0.16 | 0.47 |
| ^147^Sm | y = 4088c + 340.9 | 0.9988 | 0.33 | 0.99 |
| ^151^Eu | y = 14040c + 1354 | 0.9989 | 0.24 | 0.72 |
| ^157^Gd | y = 8230c + 318.1 | 0.9991 | 0.33 | 1.0 |
| ^159^Tb | y = 38630c + 765.0 | 0.9991 | 0.15 | 0.44 |
| ^163^Dy | y = 93880c + 339.5 | 0.9992 | 0.22 | 0.66 |
| ^165^Ho | y = 3976c + 1274 | 0.9990 | 0.018 | 0.055 |
| ^166^Er | y = 13004c + 456.6 | 0.9989 | 0.040 | 0.12 |
| ^169^Tm | y = 42960c + 683.9 | 0.9990 | 0.060 | 0.18 |
| ^172^Yb | y = 10280c + 1052 | 0.9987 | 0.22 | 0.66 |
| ^175^Lu | y = 32190c - 25.79 | 0.9991 | 0.062 | 0.19 |

Table S2. The detailed information on the pseudo-total content of REE in studied samples. SSA –sewage sludge ash; APC – air pollution control residue; FB – fuidized beds

| Type | Origin | Parameter | Sc | Y | La | Ce | Pr | Nd | Sm | Eu | Gd | Tb | Dy | Ho | Er | Tm | Yb | Lu | Total |
| --- | --- | --- | --- | --- | --- | --- | --- | --- | --- | --- | --- | --- | --- | --- | --- | --- | --- | --- | --- |
| SSA | Gdynia | Average [mg/kg] | 0.93 | 2.07 | 7.05 | 10.14 | 0.70 | 2.96 | 0.56 | 0.12 | 0.52 | 0.07 | 0.39 | 0.07 | 0.23 | 0.03 | 0.23 | 0.02 | 26.10 |
|  |  | SD [mg/kg] | 0.27 | 0.48 | 4.09 | 4.99 | 0.19 | 0.74 | 0.13 | 0.04 | 0.19 | 0.02 | 0.11 | 0.02 | 0.09 | 0.01 | 0.08 | 0.01 | 11.46 |
|  |  | Min value [mg/kg] | 0.64 | 1.60 | 3.18 | 5.85 | 0.52 | 2.21 | 0.42 | 0.08 | 0.36 | 0.05 | 0.27 | 0.05 | 0.14 | 0.02 | 0.14 | 0.02 | - |
|  |  | Max value [mg/kg] | 1.19 | 2.57 | 11.34 | 15.62 | 0.89 | 3.69 | 0.69 | 0.15 | 0.73 | 0.09 | 0.49 | 0.09 | 0.31 | 0.03 | 0.29 | 0.03 | - |
|  | Gdańsk | Average [mg/kg] | 1.87 | 3.79 | 7.61 | 13.88 | 1.30 | 5.53 | 1.08 | 0.24 | 0.89 | 0.14 | 0.76 | 0.14 | 0.41 | 0.05 | 0.39 | 0.05 | 38.14 |
|  |  | SD [mg/kg] | 0.74 | 1.10 | 2.47 | 4.71 | 0.46 | 1.97 | 0.38 | 0.08 | 0.24 | 0.04 | 0.24 | 0.04 | 0.11 | 0.01 | 0.10 | 0.01 | 12.72 |
|  |  | Min value [mg/kg] | 1.22 | 2.89 | 5.60 | 9.93 | 0.92 | 3.92 | 0.78 | 0.17 | 0.72 | 0.10 | 0.56 | 0.11 | 0.33 | 0.04 | 0.30 | 0.04 | - |
|  |  | Max value [mg/kg] | 3.49 | 6.17 | 12.99 | 24.14 | 2.32 | 9.82 | 1.93 | 0.42 | 1.40 | 0.23 | 1.27 | 0.23 | 0.65 | 0.08 | 0.59 | 0.07 | - |
|  | Łódź | Average [mg/kg] | 2.84 | 5.99 | 10.49 | 19.23 | 1.79 | 7.57 | 1.47 | 0.35 | 1.18 | 0.19 | 1.07 | 0.20 | 0.58 | 0.07 | 0.61 | 0.07 | 53.68 |
|  |  | SD [mg/kg] | 0.53 | 1.09 | 1.57 | 3.00 | 0.28 | 1.11 | 0.23 | 0.05 | 0.15 | 0.03 | 0.14 | 0.03 | 0.08 | 0.01 | 0.12 | 0.01 | 8.42 |
|  |  | Min value [mg/kg] | 2.07 | 4.39 | 8.00 | 14.79 | 1.41 | 6.07 | 1.18 | 0.28 | 1.00 | 0.16 | 0.90 | 0.17 | 0.48 | 0.06 | 0.47 | 0.06 | - |
|  |  | Max value [mg/kg] | 3.47 | 7.07 | 12.35 | 22.45 | 2.10 | 8.76 | 1.73 | 0.41 | 1.37 | 0.22 | 1.22 | 0.23 | 0.70 | 0.09 | 0.77 | 0.08 | - |
| APC | Gdańsk | Average [mg/kg] | 0.14 | 0.87 | 0.52 | 0.76 | 0.09 | 0.43 | 0.09 | 0.02 | 0.10 | 0.02 | 0.11 | 0.02 | 0.07 | 0.01 | 0.05 | 0.01 | 3.31 |
|  |  | SD [mg/kg] | 0.07 | 0.55 | 0.27 | 0.31 | 0.04 | 0.20 | 0.04 | 0.01 | 0.05 | 0.01 | 0.05 | 0.01 | 0.04 | 0.01 | 0.03 | 0.01 | 1.70 |
|  |  | Min value [mg/kg] | 0.03 | 0.14 | 0.12 | 0.21 | 0.02 | 0.10 | 0.03 | 0.01 | 0.02 | 0.01 | 0.02 | 0.01 | 0.01 | 0.01 | 0.01 | 0.01 | - |
|  |  | Max value [mg/kg] | 0.24 | 1.48 | 0.82 | 1.15 | 0.14 | 0.65 | 0.14 | 0.04 | 0.15 | 0.03 | 0.16 | 0.03 | 0.10 | 0.01 | 0.08 | 0.01 | - |
|  | Łódź | Average [mg/kg] | 0.47 | 0.96 | 1.31 | 2.53 | 0.24 | 1.04 | 0.21 | 0.05 | 0.19 | 0.03 | 0.16 | 0.03 | 0.09 | 0.01 | 0.10 | 0.01 | 7.43 |
|  |  | SD [mg/kg] | 0.12 | 0.28 | 0.33 | 0.60 | 0.06 | 0.24 | 0.05 | 0.01 | 0.05 | 0.01 | 0.03 | 0.01 | 0.02 | 0.01 | 0.04 | 0.01 | 1.86 |
|  |  | Min value [mg/kg] | 0.33 | 0.55 | 0.79 | 1.59 | 0.15 | 0.65 | 0.12 | 0.03 | 0.11 | 0.02 | 0.10 | 0.02 | 0.05 | 0.01 | 0.06 | 0.01 | - |
|  |  | Max value [mg/kg] | 0.62 | 1.30 | 1.63 | 3.18 | 0.30 | 1.27 | 0.27 | 0.06 | 0.26 | 0.04 | 0.19 | 0.04 | 0.12 | 0.01 | 0.17 | 0.01 | - |
| FB | All | Average [mg/kg] | 1.16 | 2.83 | 6.58 | 10.61 | 0.81 | 3.44 | 0.68 | 0.15 | 0.55 | 0.09 | 0.51 | 0.10 | 0.30 | 0.04 | 0.30 | 0.04 | 28.18 |
|  |  | SD [mg/kg] | 0.54 | 1.22 | 3.51 | 4.68 | 0.31 | 1.31 | 0.26 | 0.06 | 0.19 | 0.04 | 0.20 | 0.04 | 0.12 | 0.02 | 0.12 | 0.01 | 12.64 |
|  |  | Min value [mg/kg] | 0.52 | 1.33 | 2.63 | 4.83 | 0.43 | 1.83 | 0.35 | 0.08 | 0.30 | 0.04 | 0.25 | 0.05 | 0.13 | 0.02 | 0.13 | 0.02 | - |
|  |  | Max value [mg/kg] | 1.77 | 3.83 | 11.08 | 16.22 | 1.15 | 4.77 | 0.94 | 0.20 | 0.72 | 0.12 | 0.69 | 0.13 | 0.40 | 0.05 | 0.42 | 0.05 | - |

Table S3. The detailed information on the fractionation results. SSA – sewage sludge ash; FB – fluidized beds; F – fraction type, F4 – residual fraction; F3 – oxidizable fraction; F2 – reducible fraction; F1 - exchangeable and carbonates fraction

| F | Type | Parameter | Sc | Y | La | Ce | Pr | Nd | Sm | Eu | Gd | Tb | Dy | Ho | Er | Tm | Yb | Lu |
| --- | --- | --- | --- | --- | --- | --- | --- | --- | --- | --- | --- | --- | --- | --- | --- | --- | --- | --- |
| F4 | SSA | Average [mg/kg] | 2.176 | 5.013 | 9.979 | 17.541 | 1.567 | 6.647 | 1.297 | 0.283 | 1.045 | 0.166 | 0.907 | 0.172 | 0.513 | 0.065 | 0.501 | 0.060 |
|  |  | SD [mg/kg] | 0.676 | 1.549 | 1.880 | 4.501 | 0.465 | 1.975 | 0.389 | 0.080 | 0.311 | 0.047 | 0.259 | 0.049 | 0.140 | 0.018 | 0.128 | 0.017 |
|  |  | Min [mg/kg] | 0.916 | 2.110 | 7.026 | 9.420 | 0.721 | 3.054 | 0.548 | 0.137 | 0.467 | 0.077 | 0.401 | 0.078 | 0.231 | 0.029 | 0.256 | 0.027 |
|  |  | Max [mg/kg] | 3.037 | 6.892 | 13.278 | 25.536 | 2.247 | 9.492 | 1.826 | 0.390 | 1.409 | 0.225 | 1.228 | 0.232 | 0.685 | 0.088 | 0.638 | 0.079 |
| F4 | FB | Average [mg/kg] | 0.913 | 2.426 | 5.687 | 9.757 | 0.817 | 3.448 | 0.663 | 0.142 | 0.506 | 0.083 | 0.442 | 0.085 | 0.253 | 0.032 | 0.254 | 0.030 |
|  |  | SD [mg/kg] | 0.248 | 0.770 | 3.114 | 4.263 | 0.294 | 1.179 | 0.182 | 0.037 | 0.154 | 0.024 | 0.135 | 0.026 | 0.085 | 0.009 | 0.084 | 0.009 |
|  |  | Min [mg/kg] | 0.641 | 1.623 | 3.179 | 5.993 | 0.524 | 2.242 | 0.462 | 0.102 | 0.330 | 0.056 | 0.291 | 0.056 | 0.158 | 0.021 | 0.159 | 0.021 |
|  |  | Max [mg/kg] | 1.126 | 3.158 | 9.173 | 14.387 | 1.111 | 4.597 | 0.816 | 0.174 | 0.611 | 0.100 | 0.549 | 0.107 | 0.319 | 0.040 | 0.317 | 0.038 |
| F3 | SSA | Average [mg/kg] | 0.006 | <0.001 | <0.001 | <0.001 | <0.001 | <0.001 | <0.001 | <0.001 | <0.001 | <0.001 | <0.001 | <0.001 | <0.001 | <0.001 | <0.001 | <0.001 |
|  |  | SD [mg/kg] | - | - | - | - | - | - | - | - | - | - | - | - | - | - | - | - |
|  |  | Min [mg/kg] | 0.006 | <0.001 | <0.001 | <0.001 | <0.001 | <0.001 | <0.001 | <0.001 | <0.001 | <0.001 | <0.001 | <0.001 | <0.001 | <0.001 | <0.001 | <0.001 |
|  |  | Max [mg/kg] | 0.007 | <0.001 | <0.001 | <0.001 | <0.001 | <0.001 | <0.001 | <0.001 | <0.001 | <0.001 | <0.001 | <0.001 | <0.001 | <0.001 | <0.001 | <0.001 |
| F3 | FB | Average [mg/kg] | 0.006 | <0.001 | <0.001 | <0.001 | <0.001 | <0.001 | <0.001 | <0.001 | 0.002 | <0.001 | <0.001 | <0.001 | <0.001 | <0.001 | <0.001 | <0.001 |
|  |  | SD [mg/kg] | - | - | - | - | - | - | - | - | - | - | - | - | - | - | - | - |
|  |  | Min [mg/kg] | 0.006 | <0.001 | <0.001 | <0.001 | <0.001 | <0.001 | <0.001 | <0.001 | <0.001 | <0.001 | <0.001 | <0.001 | <0.001 | <0.001 | <0.001 | <0.001 |
|  |  | Max [mg/kg] | 0.006 | <0.001 | <0.001 | <0.001 | <0.001 | 0.002 | <0.001 | <0.001 | 0.004 | <0.001 | <0.001 | <0.001 | <0.001 | <0.001 | <0.001 | <0.001 |
| F2 | SSA | Average [mg/kg] | 0.014 | 0.006 | 0.010 | 0.016 | 0.002 | 0.006 | 0.002 | <0.001 | 0.002 | <0.001 | <0.001 | <0.001 | <0.001 | <0.001 | <0.001 | <0.001 |
|  |  | SD [mg/kg] | 0.007 | 0.007 | 0.011 | 0.018 | 0.001 | 0.007 | 0.001 | - | 0.002 | - | 0.001 | - | 0.001 | - | 0.001 | - |
|  |  | Min [mg/kg] | 0.006 | <0.001 | 0.002 | 0.003 | <0.001 | <0.001 | <0.001 | <0.001 | <0.001 | <0.001 | <0.001 | <0.001 | <0.001 | <0.001 | <0.001 | <0.001 |
|  |  | Max [mg/kg] | 0.026 | 0.027 | 0.039 | 0.066 | 0.005 | 0.024 | 0.005 | <0.001 | 0.005 | <0.001 | 0.004 | <0.001 | 0.003 | <0.001 | 0.004 | <0.001 |
| F2 | FB | Average [mg/kg] | 0.012 | 0.017 | 0.024 | 0.027 | 0.002 | 0.009 | 0.002 | <0.001 | 0.002 | <0.001 | 0.002 | <0.001 | <0.001 | <0.001 | <0.001 | <0.001 |
|  |  | SD [mg/kg] | 0.011 | 0.024 | 0.028 | 0.028 | 0.002 | 0.009 | 0.001 | - | 0.002 | - | 0.001 | - | 0.001 | - | - | - |
|  |  | Min [mg/kg] | 0.006 | <0.001 | 0.003 | 0.003 | <0.001 | <0.001 | <0.001 | <0.001 | <0.001 | <0.001 | <0.001 | <0.001 | <0.001 | <0.001 | <0.001 | <0.001 |
|  |  | Max [mg/kg] | 0.025 | 0.044 | 0.056 | 0.057 | 0.004 | 0.019 | 0.004 | <0.001 | 0.005 | <0.001 | 0.003 | <0.001 | 0.002 | <0.001 | 0.002 | <0.001 |
| F1 | SSA | Average [mg/kg] | 0.024 | 0.003 | 0.003 | 0.003 | <0.001 | <0.001 | <0.001 | <0.001 | <0.001 | <0.001 | <0.001 | <0.001 | <0.001 | <0.001 | <0.001 | <0.001 |
|  |  | SD [mg/kg] | 0.008 | 0.001 | 0.002 | 0.002 | - | 0.001 | - | - | - | - | - | - | - | - | - | - |
|  |  | Min [mg/kg] | 0.009 | <0.001 | <0.001 | <0.001 | <0.001 | <0.001 | <0.001 | <0.001 | <0.001 | <0.001 | <0.001 | <0.001 | <0.001 | <0.001 | <0.001 | <0.001 |
|  |  | Max [mg/kg] | 0.041 | 0.004 | 0.007 | 0.007 | <0.001 | 0.003 | <0.001 | <0.001 | <0.001 | <0.001 | <0.001 | <0.001 | <0.001 | <0.001 | <0.001 | <0.001 |
| F1 | FB | Average [mg/kg] | 0.012 | 0.003 | 0.002 | 0.002 | <0.001 | <0.001 | <0.001 | <0.001 | <0.001 | <0.001 | <0.001 | <0.001 | <0.001 | <0.001 | <0.001 | <0.001 |
|  |  | SD [mg/kg] | 0.010 | 0.001 | 0.000 | 0.001 | - | - | - | - | - | - | - | - | - | - | - | - |
|  |  | Min [mg/kg] | 0.006 | 0.002 | <0.001 | <0.001 | <0.001 | <0.001 | <0.001 | <0.001 | <0.001 | <0.001 | <0.001 | <0.001 | <0.001 | <0.001 | <0.001 | <0.001 |
|  |  | Max [mg/kg] | 0.025 | 0.004 | 0.002 | 0.003 | <0.001 | <0.001 | <0.001 | <0.001 | <0.001 | <0.001 | <0.001 | <0.001 | <0.001 | <0.001 | <0.001 | <0.001 |
